# Co‐design of dementia prevention program for Aboriginal Australians (DAMPAA)

**DOI:** 10.1002/alz.13032

**Published:** 2023-03-18

**Authors:** Irene Mateo‐Arriero, Alexander Lalovic, Glennette Dowden, Lesley Markey, Kay L. Cox, Leon Flicker, Dawn Bessarab, Sandra Thompson, Carmel Kickett, Deborah Woods, Carmela F. Pestell, Paula Edgill, Christopher Etherton‐Beer, Kate Smith

**Affiliations:** ^1^ Centre for Aboriginal Medical and Dental Health University of Western Australia Perth WA Australia; ^2^ West Australian Centre for Health and Ageing University of Western Australia Perth WA Australia; ^3^ Western Australian Centre for Rural Health University of Western Australia Geraldton WA Australia; ^4^ Moorditj Koort Aboriginal Corporation Medina WA Australia; ^5^ Geraldton Regional Aboriginal Medical Service Geraldton WA Australia; ^6^ School of Psychological Science University of Western Australia Perth WA Australia; ^7^ Derbarl Yerrigan Health Service East Perth WA Australia

**Keywords:** dementia, early intervention, geriatrics, health promotion, indigenous, program co‐design, risk management, theory of change

## Abstract

**Introduction:**

Dementia is highly prevalent in older Aboriginal Australians, with several modifiable risk factors. Currently, there is limited evidence on how to prevent cognitive decline in Aboriginal Australians.

**Methods:**

Based on our Theory of Change (ToC) framework, we co‐developed the Dementia risk management and prevention program for Aboriginal Australians (DAMPAA) aged over 45 years in partnership with Aboriginal community‐controlled organizations (ACCOs) and Elders. Qualitative data were collected through ACCO staff workshops, Elders yarning, and governance groups to inform the protocol. Additionally, we conducted a small pilot study.

**Results:**

Expected DAMPAA ToC outcomes are: (1) improved daily function, (2) better cardiovascular risk management, (3) falls reduction, (4) improved quality of life, and (5) reduced cognitive decline. Attendance enablers are social interaction, environment, exercise type/level, and logistics.

**Discussion:**

Findings suggest that ToC is an effective collaborative approach for co‐designing Aboriginal health programs.

## 1 BACKGROUND

Aboriginal and Torres Strait Islander people (hereafter respectfully referred to as Aboriginal^1^) are Australia's First Peoples and the longest continuous culture in the world, with deep continuing spiritual connections with ancestral lands. Improving the health and wellbeing of Aboriginal peoples is a key Australian government policy goal.^2^ Older Aboriginal people have a vital role in the health and wellbeing of communities. Elders hold and teach cultural knowledge, care for country and family, and are important role models for younger generations.^3^, ^4^ The high prevalence of dementia in this population^5^, ^6^, ^7^ and resulting functional limitations are a substantial concern to older Aboriginal peoples and communities,^4^ and the health care system.^8^ Maintaining Elders’ ability to fulfill their eldership role through effective dementia prevention programs is a research priority identified by Aboriginal communities and organizations.^9^, ^10^

A study of First Nations people in Queensland found that 52% of dementia burden could be attributed to 11 potentially modifiable factors (e.g., physical inactivity, social isolation),^11^ previously shown to be dementia risk factors in other Aboriginal communities.^6^, ^7^, ^12^ Physical inactivity is the third leading risk factor for disease burden in Aboriginal Australians.^13^ Regular physical activity in mid‐ to late life significantly reduces risk of cognitive decline in older people,^14^, ^15^ delays disability,^16^ prolongs independent living,^17^ and is associated with better quality of life.^18^

Cardiovascular disease and dementia have common risk factors and pathophysiology.^19^ A systematic review of Australian randomized controlled trials (RCTs) of cardiovascular interventions in Aboriginal communities^20^ found the intervention with greatest health impact was the multimodal community‐based Looma Healthy Lifestyle program.^21^ This program involved sport, walking groups, and risk‐factor education sessions. Health programs run by Aboriginal community‐controlled organizations (ACCOs) involving education and exercise can significantly improve specific health outcomes and quality of life.^22^, ^23^ Group and community activities centered on social interaction are preferred formats of physical activity for Aboriginal people.^24^, ^25^ Cultural safety of the environment where physical activity occurs and the provision of appropriate, relevant health education are also important.^24^, ^25^ Despite mixed results, current evidence from large‐scale RCTs points to multi‐domain, complex programs (e.g., FINGER, Pre‐DIVA) which target multiple risk factors as the most effective prevention approach.^26^, ^27^ Increasingly, there is advocacy for mid‐life dementia prevention programs, given the emerging evidence that mid‐life hypertension, obesity, and diabetes are associated with increased dementia risk.^26^, ^28^, ^29^

Despite what is known about dementia in the Aboriginal community, evidence on programs showing how to improve cognitive functioning in Aboriginal peoples is lacking. The Dementia Prevention and Risk Management Program for Aboriginal Australians (DAMPAA) study is a targeted, culturally appropriate, Aboriginal Health Practitioner (AHP)‐coordinated, risk‐factor management program. DAMPAA aims to reduce cognitive decline and functional impairment in Aboriginal Australians aged 45 years and over at risk of dementia, and evaluate its effectiveness through an RCT.^30^

**RESEARCH IN CONTEXT**

1. **Systematic Review**: The authors used established sources to review existing literature describing dementia rates and risk factors for Aboriginal Australians and dementia prevention programs developed for the general population. Theory of Change frameworks used to design and evaluate complex health programs were also reviewed. These publications are appropriately cited.
2. **Interpretation**: Our findings support an approach which engaged community members, participants, and stakeholders in using a Theory of Change framework in research to design, implement and evaluate Aboriginal health programs. Extensive community consultation helped ensure the cultural content, practices and values of Aboriginal people were respected and upheld.
3. **Future Directions**: The DAMPAA program will be evaluated through a randomized controlled trial and parallel process evaluation based on the ToC framework developed in this study. Researchers working within Aboriginal health contexts will be able to replicate this approach to co‐design programs for and with Aboriginal communities.

This article describes the process of developing the DAMPAA program using a Theory of Change (ToC) approach. ToC was chosen to guide development and evaluation of the protocol, as it is recommended for evaluation of complex, multi‐domain programs.^31^ A ToC is developed through key stakeholder involvement and community consultation.^31^ Community engagement is particularly important in Aboriginal health research.^32^ The ToC development approach ensures the community, service providers, and key stakeholders shape the development of DAMPAA at all stages of the research process, enabling a more effective translation into sustainable outcomes.^33^ A process evaluation (based on the ToC) will run in parallel to the RCT to inform and refine the implementation of DAMPAA.^34^

This article outlines the co‐design process of developing the DAMPAA ToC and subsequent program protocol. Our aim is to illustrate a method for co‐designing a prevention program with (and for) the Aboriginal community through the ToC approach. Making this knowledge available to researchers and health services working with Aboriginal communities may assist development of future programs.

## 2 METHOD

### 2.1 Design

The DAMPAA program was designed using Indigenous research methodologies, where the views, experiences, and recommendations of Aboriginal participants and Aboriginal community‐controlled health service providers are the central data source. Together with a ToC framework,^31^ this approach was adopted to actively engage community members, participants, stakeholders, and Aboriginal and non‐Aboriginal researchers in program design and evaluation. Ethics approval was obtained from Western Australian Aboriginal Health Ethics Committee (#867) and the University of Western Australia Human Research Ethics Committee (RA/4/20/4944).

#### 2.1.1 Theory of Change framework

ToC is an approach to program design and evaluation aiming to understand how and why a program works.^35^ A ToC describes causal pathways through which a program is hypothesized to effect change, outlining outcomes necessary to achieve the desired impact, using indicators to track progress.^36^ Activities needed to progress through various outcomes are mapped onto the ToC's causal pathway. ToC also includes assumptions, the conditions required for the program to work.^36^ Our approach to ToC development consisted of a combination of learning from existing research and stakeholder workshops. This ToC may evolve as the program is delivered to multiple cohorts. Assumptions, indicators, and outcomes may be added or removed through ongoing evaluation.

### 2.2 Consultation with Elders groups

The DAMPAA team involved Aboriginal Elders in developing the program and its implementation protocol through consulting an Elders governance group (one man, five women) and an Elders community aged care group at Marmum Yorga (four men, four women) in February 2019. The Elders governance group was formed in 2016 to assist previous research. In 2019 they agreed to support cultural governance of DAMPAA. Several consultation sessions were conducted and aimed to ascertain barriers and enablers of change (ToC's assumptions), specific considerations, and general interest/demand for a program of this kind. Consultation sessions used yarning, an appropriate and culturally safe method for communication with Aboriginal Australians,^37^ for discussion and gathering of information. Yarning is led by the researcher and participants are encouraged to share their story. This method builds trust and connection between the parties.^38^, ^39^ Researchers began with social yarning to establish a connection with Elders, followed by research yarning with open questions specific to the data being collected. These sessions ensured the cultural content, practices and values of older Aboriginal people, their families and community were respected and upheld. The second half of the Elders community group session was divided into women's and men's yarning groups.

### 2.3 Establishing partnerships with Aboriginal community‐controlled organizations

In our initial ToC framework, a key component is “service provider buy‐in”. This step is crucial to program success, translation, and implementation into the wider health system.^35^ Thus, it was important for the DAMPAA team to develop mutually beneficial partnerships with ACCOs. We partnered with three organizations: Derbarl Yerrigan Aboriginal Health Service (Perth WA), Moorditj Koort Aboriginal Corporation (Perth WA), and Geraldton Regional Aboriginal Medical Service (Geraldton WA). As part of these partnerships, the Chief Investigator (CI) group incorporated an ACCO representative from each site. Additionally, we held meetings to discuss the partnership relationships, identify current protocols for people with dementia within health services, how we could build on these strengths, and what the research team could offer organizations.

#### 2.3.1 Service provider workshops

Three half‐day workshops were conducted between July 2018 and March 2019 with 24 (eight male) senior staff and site managers working in ACCOs. Workshops evaluated enablers and challenges at each stage of Aboriginal communities’ engagement with a new health and wellbeing program. Workshops included small‐group exercises, brainstorming activities, and open discussions. At the end of each workshop, the information obtained was transcribed and workshop minutes recorded. This information was used to compile a comprehensive list of enablers and challenges of change, and important practical considerations for implementing DAMPAA locally. These were used in refining the DAMPAA ToC and program protocol. Service provider involvement also aimed to improve the feasibility and translation of DAMPAA.

### 2.4 Pilot study

In November 2019, after comprehensive consultation with stakeholders, Elders, and community members, the DAMPAA team conducted a pilot study to assess engagement and feasibility of the program implementation in community organizations.

#### 2.4.1 Participants

Participants were recruited through Derbarl Yerrigan Health Service East Perth (Derbarl Yerrigan) and provided written informed consent before undergoing an initial screening to determine eligibility. Screening included the KICA‐Cognitive assessment^40^ to identify participants with cognitive impairment not dementia. Ten participants (7 female) aged 45 to 81 years (M = 63.5, SD = 11.7) were recruited via community outreach (e.g., attending community events, holding information sessions at ACCOs) for the pilot study. They were randomized to the DAMPAA (*n*  =  5, three female) or control group (*n*  =  5, four female). Participants in the control condition completed the same initial assessments but did not undertake the program, they maintained their usual routine. One participant from the DAMPAA group withdrew from the study at week 1 due to family commitments. The pilot study was suspended at week 16 (March 2020) and subsequently ceased due to the coronavirus disease 2019 (COVID‐19) outbreak.

#### 2.4.2 Materials

Each participant was provided with a file, containing education session handouts, resources and worksheets, and the home exercise sessions handout and exercise calendar. AHPs and exercise specialists were involved in developing and adapting program materials, which were then reviewed by project management.

##### 2.4.2.1 Education session handouts, resources, and worksheets

Handouts created specifically for DAMPAA corresponded to each of the six pilot education session topics: (1) introduction to DAMPAA, (2) exercise and brain health, (3) good tucker – nutrition, (4) diabetes prevention/management, (5) medication management, and (6) falls prevention (only sessions 1‐3 were delivered prior to pilot ceasing).

##### 2.4.2.2 Home exercise sessions handout and calendar

Each participant was provided with a weekly home exercise session handout that included pictures and text descriptions for each exercise and a monthly exercise calendar. Participants filled this out each week when they completed a session and reported their rate of perceived exertion. Participants used the exercise session handouts to complete one home exercise session per week.

#### 2.4.3 Measures

The participant exit interview schedule was loosely based on principles from the Clients’ Perception of the Quality of Chronic Condition Care Survey.^41^ It included questions regarding satisfaction (e.g., Did you enjoy the DAMPAA group exercise sessions?), and program appropriateness (e.g., Did you think that the exercise program was suitable for your own health and body?). The interview included questions regarding preferences for aspects of the program (e.g., ‘What did you enjoy most about the study?’). Participants’ responses were captured through notetaking by AHPs. The exit interview schedule captured participants’ self‐reported changes in areas of functioning (e.g., strength, memory etc.) over the past 6 months. Last, it included a question regarding impact of COVID‐19 on participants’ exercise levels.

#### 2.4.4 Procedure

Participants who were eligible and interested in taking further part underwent an additional two baseline assessment sessions before being randomized into the DAMPAA group or Usual Care group. General practitioner (GP) clearance was obtained for each participant prior to randomization. The DAMPAA program was delivered twice per week at Derbarl Yerrigan until week 16. Participants were withdrawn from the pilot study and phone exit interviews were conducted by AHPs.

### 2.5 Data analysis

Data analysis was conducted by first author (I.M.A.), a non‐Aboriginal researcher. Thematic analysis, using qualitative analysis software NVivo, was used to analyze qualitative data gathered to develop the DAMPAA program protocol.^42^ Co‐author (L.M.), an Aboriginal researcher, cross‐checked the data coding and interpretation for accuracy. Self‐reported quantitative data collected during exit interviews were analyzed using Microsoft Excel. Results were used to refine the initial DAMPAA ToC and presented to the Elders governance group for review before finalizing the program protocol and the RCT commencing.

## 3 RESULTS

### 3.1 Theory of Change

**FIGURE 1:**
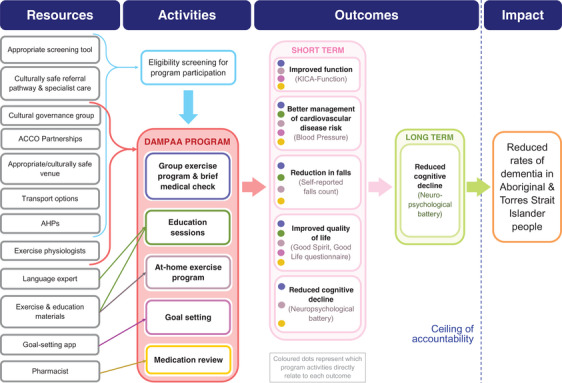
DAMPAA Theory of Change diagram.

The initial DAMPAA ToC (Figure 1) identifies the resources, activities, assumptions, outcomes (long and short‐term), indicators, and impact proposed for a systematic approach to dementia prevention.

The DAMPAA ToC includes 10 assumptions not shown in the diagram, listed hereafter. There are three assumptions within the links between resources and activities: (1) screening tool used is valid, (2) AHPs are available for recruitment/running the program, and (3) AHPs and exercise physiologists can appropriately run the program adhering to the protocol. Assumptions which need to be met in order for DAMPAA activities to produce the desired outcomes are: (4) sufficient attendance, (5) ability and confidence to come together safely for the group exercise program (hindered during pilot due to COVID‐19 restrictions), (6) having the time and safe environment for at‐home exercises, (7) appropriate exercise level for participants, (8) appropriate content and delivery of education sessions, (9) GPs enacting changes in medication based on pharmacist review, and (10) setting achievable goals that participants take action to reach.

### 3.2 Qualitative findings on enablers and barriers to program attendance

#### 3.2.1 Social interaction

The Elders community group and the four DAMPAA pilot participants emphasized social connection during the program as crucial, for example “create a social aspect to the activity” (men's yarning group). Some comments during the exit interviews were: “meet other people, get social contact, enjoy” and, when answering the question ‘What did you enjoy most about the study?’, they replied “Meeting new people”. This emphasized the importance of the group social aspect of DAMPAA.

#### 3.2.2 Environment

Having a suitable space to run the program emerged in each Elders’ group, and during participant exit interviews. This related to an appropriate venue to conduct the group program, and each participant having a suitable and safe space to complete their at‐home exercise. Both Elders and participants mentioned potential difficulties completing the at home program with family at home, particularly with extended family staying with them. Participants talked about the environment, outdoor and indoor spaces, being in a “friendly” environment, and having a nature setting (e.g., park). This idea of the '‘environment’' of the program is captured in both the resources and assumptions of the DAMPAA ToC.

#### 3.2.3 Exercise

From the Elders’ groups, the possibility of including special exercises (e.g., water‐aerobics) or other types of activities which are social and active was discussed. Feedback from participant exit interviews indicated the level of exercise in the program was appropriate (e.g., “…felt that she could do. The exercises not hard to do”). This finding provides evidence that assumption seven of the DAMPAA ToC (appropriate exercise level) was met.

#### 3.2.4 Logistics

Other findings include logistical aspects of the program, such as the importance of scheduling sessions to fit in with family commitments such as caring for grandchildren, a consideration mentioned by both service providers and Elders. Offering creche services was suggested during a service‐provider workshop. Other logistical considerations were having suitable transport options, role of physical health (i.e., being injury‐free, feeling well), and participants’ motivation to attend the program, which was positively influenced by the social accountability of being part of the group.

### 3.3 DAMPAA program protocol

Based on the DAMPAA ToC, findings from ToC workshops and pilot study, the final DAMPAA protocol (briefly described below) was developed.

Participants undertake a 12‐month program and can withdraw at any time. DAMPAA consists of two periods, action (1‐6 months) and maintenance (7‐12 months). The action period consists of face‐to‐face group exercise and education delivered by DAMPAA staff at an ACCO. Participants attend two group exercise sessions per week and complete one at home. Sessions consist of walking, strength and balance exercises, and stretching. Elders recommended activities such as Tai‐Chi and water aerobics as alternative one‐off sessions to expose interested participants to different modes of physical activity and link to available community programs. Personalized goal setting is completed in the first 2 weeks of the program and reviewed every 4 weeks. Education sessions are delivered monthly in a group yarning style after an exercise session, starting in week 5 and incorporating learning Noongar language, nutrition, and wellbeing. The Noongar language was added as a recommendation by our Elders advisory group. Elders indicated this would be a way for participants to connect with culture and benefit brain and spiritual health. A pharmacist conducts medication reviews during the action period.

Following the action period, participants complete a 6‐month follow‐up assessment. The maintenance period consists of three home‐based exercise sessions per week. DAMPAA staff monitor progress via weekly telephone calls. Following the maintenance period, participants complete a final 12‐month assessment.

## 4 DISCUSSION

This article illustrates the co‐design process for a dementia prevention program with (and for) the Aboriginal community using a ToC approach. The ToC development approach consisted of a combination of learning from existing research and community consultation. The extensive consultation process aimed to engage community members, participants, and stakeholders in program design, helping to ensure the cultural content, practices and values of Aboriginal people were respected. The goal is that this existing knowledge can be accessed by researchers and health organizations working with Aboriginal communities to aid in creating and developing future programs.

Other studies in Aboriginal health have used similar approaches to develop their program protocols, for example Healing the Right Way (HRW)^34^ incorporates an Aboriginal research framework drawing from Indigenous Standpoint Theory. While they did not use ToC explicitly to develop the trial protocol, their approach was similar, including partnerships with ACCOs, and consultation guided by an Aboriginal Reference Group.^34^ They too planned a process evaluation of the HRW program, albeit using the Consolidated Framework for Implementation Research (CFIR)^43^ instead of ToC.^34^ In terms of process evaluation, the main difference is that ToC begins with program co‐development and continues as a method for process evaluation, whereas CFIR focuses only on the latter.^31^, ^43^ We decided the ToC approach was better suited to DAMPAA as, unlike CFIR, it enabled use of the same framework from initial program development through to the process evaluation stage of implementation.

Another study in the area of Aboriginal health using a similar approach was “Let's CHAT.”^33^ They aimed to implement and evaluate a culturally responsive best practice model of care for Aboriginal people with cognitive impairment and/or dementia.^33^ Let's CHAT resembles DAMPAA and HRW in using a co‐design collaboration with ACCOs and communities to develop their model of care^33^ and used the integrated Promoting Action on Research Implementation in Health Services (i‐PARIHS) framework for program development, implementation and process evaluation.^33^, ^44^ While the three approaches (ToC, CFIR, and i‐PARIHS) may appear quite different, they share the same core principles with regard to process evaluation: (1) involvement of community, service providers, and key stakeholders; (2) continuous feedback and refinement of protocol; and (3) accounting for contextual factors which may impact program outcomes.^31^, ^43^, ^44^

Previously, ToC frameworks have been used to evaluate multi‐domain, complex interventions,^31^ which, together with the focus on stakeholder involvement, was the reason why this approach was used. A strength of the current study is the detailed account of methods used and the process undergone to create the DAMPAA ToC and program protocol. While there are various methods used to develop ToCs in the literature,^31^ Breuer and colleagues (2015) state that most studies do not report enough detail about the process of developing a ToC, and how ToC was used in program design. This lack of detailed reporting prevents other researchers from harnessing existing knowledge, and leads to slower progress.^31^ This study addressed this deficit by comprehensively reporting methods used in creating the ToC and DAMPAA protocol.

The current study has several limitations. First, there was a greater proportion of women than men in this study, which could affect the findings. However, we did not observe significant differences in the enablers and challenges discussed in the Elders groups, service provider workshops and by the program participants, which suggests these findings were common across all data sources. Furthermore, we did not find significant differences in the enablers and challenges discussed in the Elders community group sessions when divided into women's and men's yarning groups. Last, it is important to mention that due to the impact of COVID‐19 on research capacity, some ACCOs involved in the development of the ToC and protocol declined ongoing involvement in the implementation. Therefore, the RCT will be conducted in sites not engaged in protocol consultations. However, through the program evaluation process, any site‐specific contextual factors which influence program outcomes can be identified.

In conclusion, ToC is an effective collaborative method for co‐designing Aboriginal health programs. This approach was used to develop the DAMPAA ToC and subsequent program protocol. DAMPAA will be evaluated through an RCT and parallel process evaluation based on indicators and outcomes outlined in the ToC. In future, researchers working within Aboriginal health contexts will be able to replicate this methodology to co‐design programs for and with Aboriginal communities.

## AUTHOR CONTRIBUTIONS

Conceived and designed the research: Smith, Flicker, Cox, Thompson, Bessarab, Etherton‐Beer, Pestell. Performed the research activities: Smith, Cox, Lalovic, Dowden, Pestell.

Managed recruitment for ACCO staff workshops: Edgill, Kickett, Woods. Analyzed the data: Mateo‐Arriero, Markey. Drafted the manuscript: Mateo‐Arriero, Lalovic, Smith. Other: all authors reviewed the manuscript for important intellectual content and approved its submission for publication.

## CONFLICT OF INTEREST STATEMENT

All authors declare that they have no conflicts of interest to disclose. Author disclosures are available in the supporting information.

## CONSENT STATEMENT

All human participants provided written informed consent to participate in the pilot study.

## Supporting information

Supporting Information
